# Supplementary material for: Gaps in the Type 1 Diabetes Mellitus care cascade: a national perspective using South Africa’s National Health Laboratory Service (NHLS) database
Source: medRxiv. 2025 Oct 21:2025.09.26.25336712. Originally published 2025 Sep 28. Preprint. [Version 2] doi: 10.1101/2025.09.26.25336712 (PMC12486024; doi:10.1101/2025.09.26.25336712)
Supplement: Supplement 1 [file media-1.docx]

**Supplemental Figure 1a-1f. Quarterly glucose (random or fasting) and HbA1c lab events nationally and stratified by HIV status and facility type**.

**
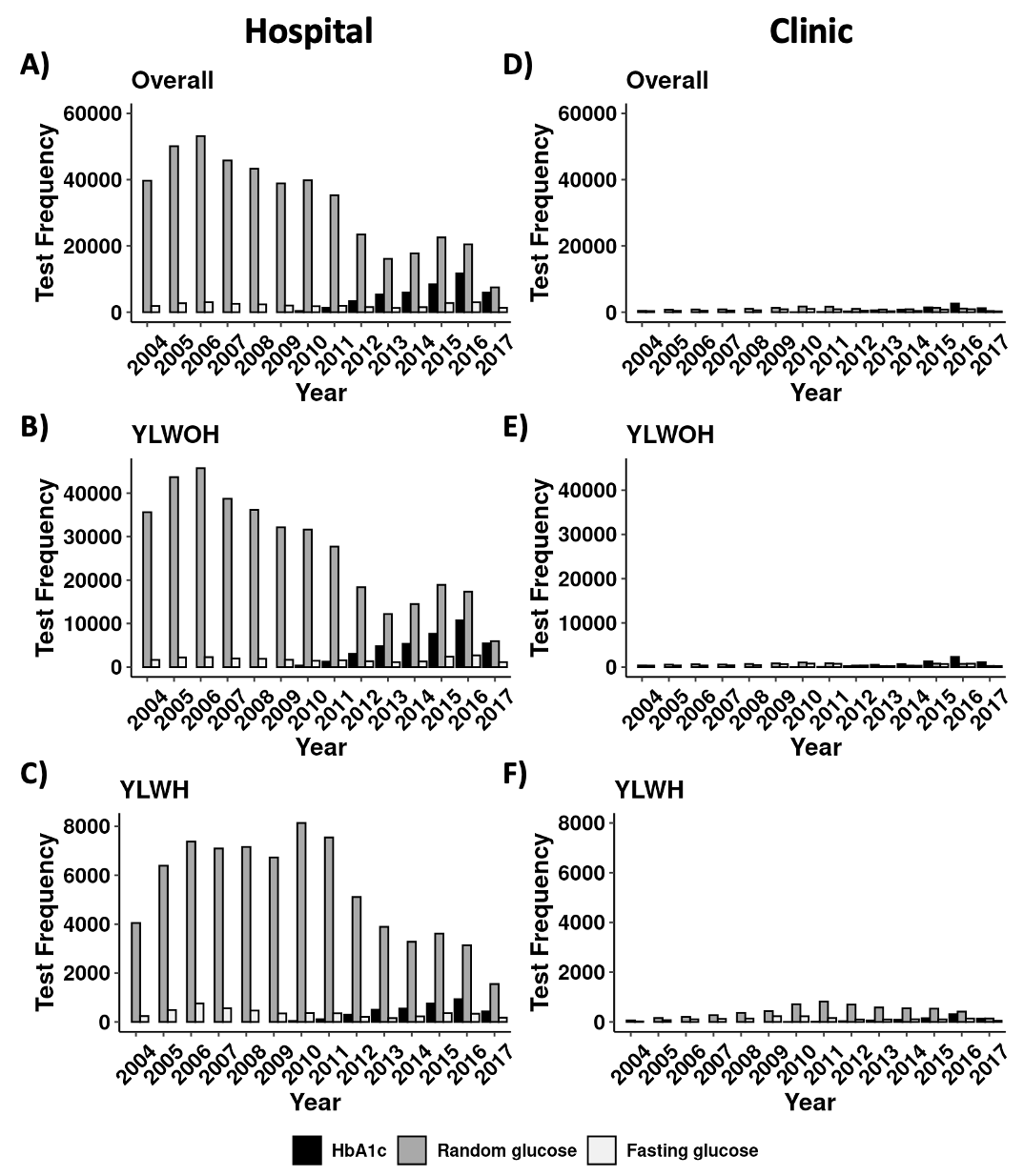
**

*YLWH – youth living with HIV; YLWOH youth living without HIV
